# Mosquito Vector Diversity across Habitats in Central Thailand Endemic for Dengue and Other Arthropod-Borne Diseases

**DOI:** 10.1371/journal.pntd.0002507

**Published:** 2013-10-31

**Authors:** Panpim Thongsripong, Amy Green, Pattamaporn Kittayapong, Durrell Kapan, Bruce Wilcox, Shannon Bennett

**Affiliations:** 1 Department of Tropical Medicine, Medical Microbiology, and Pharmacology, University of Hawai'i at Manoa, Honolulu, Hawai'i, United States of America; 2 Department of Microbiology, California Academy of Sciences, San Francisco, California, United States of America; 3 Department of Microbiology, University of Hawai'i at Manoa, Honolulu, Hawai'i, United States of America; 4 Center of Excellence for Vectors and Vector-Borne Diseases, Faculty of Science, Mahidol University at Salaya, Nakhon Pathom, Thailand; 5 Department of Entomology and Center for Comparative Genomics, California Academy of Sciences, San Francisco, California, United States of America; 6 Center for Conservation Research Training, Pacific Biosciences Research Center, University of Hawai'i at Manoa, Honolulu, Hawai'i, United States of America; 7 Integrative Research and Education Program, Faculty of Public Health, Mahidol University, Bangkok, Thailand; Tropical Disease Research Laboratory, Faculty of Medicine, Khon Kaen University, Khon Kaen, Thailand; Centers for Disease Control and Prevention, Puerto Rico, United States of America

## Abstract

Recent years have seen the greatest ecological disturbances of our times, with global human expansion, species and habitat loss, climate change, and the emergence of new and previously-known infectious diseases. Biodiversity loss affects infectious disease risk by disrupting normal relationships between hosts and pathogens. Mosquito-borne pathogens respond to changing dynamics on multiple transmission levels and appear to increase in disturbed systems, yet current knowledge of mosquito diversity and the relative abundance of vectors as a function of habitat change is limited. We characterize mosquito communities across habitats with differing levels of anthropogenic ecological disturbance in central Thailand. During the 2008 rainy season, adult mosquito collections from 24 sites, representing 6 habitat types ranging from forest to urban, yielded 62,126 intact female mosquitoes (83,325 total mosquitoes) that were assigned to 109 taxa. Female mosquito abundance was highest in rice fields and lowest in forests. Diversity indices and rarefied species richness estimates indicate the mosquito fauna was more diverse in rural and less diverse in rice field habitats, while extrapolated estimates of true richness (Chao1 and ACE) indicated higher diversity in the forest and fragmented forest habitats and lower diversity in the urban. *Culex* sp. (Vishnui subgroup) was the most common taxon found overall and the most frequent in fragmented forest, rice field, rural, and suburban habitats. The distributions of species of medical importance differed significantly across habitat types and were always lowest in the intact, forest habitat. The relative abundance of key vector species, *Aedes aegypti* and *Culex quinquefasciatus*, was negatively correlated with diversity, suggesting that direct species interactions and/or habitat-mediated factors differentially affecting invasive disease vectors may be important mechanisms linking biodiversity loss to human health. Our results are an important first step for understanding the dynamics of mosquito vector distributions under changing environmental features across landscapes of Thailand.

## Author Summary

Biodiversity affects the long-term health of a community by virtue of the many interactions constituent organisms depend upon. Mosquito-borne diseases are particularly likely to respond to changes on multiple transmission levels that span mosquito and vertebrate host communities. We characterized mosquito communities across habitats with differing levels of anthropogenic degradation in central Thailand. During the 2008 rainy season, 83,325 adult mosquitoes were collected from 24 sites, representing 6 habitat types ranging from forest to urban, of which 62,126 females were assigned to 109 taxa. Extrapolated estimates of true richness (Chao1 and ACE) indicated higher diversity of mosquito communities in forest/fragmented forests and lower diversity in urban habitats. Species of medical importance differed significantly across habitats and were always lowest in forest. The relative abundance of vectors *Aedes aegypti* and *Culex quinquefasciatus* was negatively correlated with biodiversity, suggesting that direct species interactions and/or habitat-mediated factors differentially affecting invasive vectors may be important mechanisms linking biodiversity loss to human health. Our results represent an important first step towards understanding the distributions of mosquitoes including disease vectors under changing environmental features.

## Introduction

Our expanding and increasingly globalized human population has seen the emergence of new infectious diseases such as SARS and the resurgence of familiar diseases such as dengue and influenza to epidemic proportions. At the same time, our environment has experienced substantial ecological disturbance due to habitat destruction, invasive species and climate change, with dramatic losses of native species and ecosystems. Biodiversity, or the variety of life forms and functions in nature [1], affects the stability and long-term health of communities by virtue of rich and life-sustaining networks of ecological and evolutionary interactions. Changes in biodiversity have the potential to affect the risk of infectious diseases in a system by disrupting normal relationships between hosts and pathogens. Bonds et al. [2] report that biodiversity loss is an important factor in the increase of vector-borne and parasitic diseases, which in turn have negative economic and human health impacts. This has been demonstrated experimentally with reduced infection intensities of the human parasite *Schistsoma mansoni* in diverse snail communities [3]. Anthropogenic changes specifically have been linked to the recent emergence of certain infectious diseases [4], [5]. For example, in Malaysia the emergence of Nipah virus has been linked to agricultural intensification [6]. In Australia, urban habituation increased the number of fruit bats in contact with humans and domestic animals, resulting in the emergence of Hendra virus [7]. In the eastern United States, forest fragmentation and urbanization led to reduced host diversity, allowing disease-competent rodent hosts to dominate the community, contributing to the emergence of Lyme disease [8]. Thus, in these and many other cases, anthropogenic environmental changes disturb ecological relationships in communities and consequently affect the distribution and relative abundance, or biodiversity, of organisms involved in disease transmission.

The mechanisms by which anthropogenic habitat change can lead to biodiversity loss include changes in the relative abundance of species already present in a community, the introduction of new species, or both, where changes in all species may be brought about by direct or indirect interactions (e.g., competition, predation or a change in resources, respectively). The specific mechanisms by which biodiversity change affects infectious disease distribution depend on the biology of the pathogen and could include the loss of alternate and less competent hosts (the dilution effect [9], [10]), the breakdown of ecological barriers that normally check transmission including cross-species transmission to new hosts, or the generation of new ecological niches in which certain pathogens can flourish (reviewed in [9]). The introduction of invasives in particular can directly, through the introduction of specific pathogens and their role as an optimal niche, or indirectly, through disrupting other species' ecological relationships, contribute to changing infectious disease distribution. For a vector-borne disease system, which integrates multiple trophic levels across communities, biodiversity change may involve the shifting of overall vector community feeding patterns [8], [11], [12], vector distribution, density, activity and longevity, thereby altering host exposure to vector populations, and hence disease risk [13], [14], [15], [16], [17]. The introduction of human-adapted vectors can both introduce new human pathogens as well as reduce the relative abundance of other species, or their relationships to hosts, leading to biodiversity loss and changes in infectious disease distribution.

In this study, we assess variation in the biodiversity of mosquito communities that include many types of vectors and potential pathogens across habitats differentially impacted by humans, to address when and how these mosquito communities change, as an important first step in identifying potential mechanisms by which this change might affect host-vector interactions and ultimately vector-borne disease risk. Biodiversity of mosquito communities may change across landscapes through multiple mechanisms, including changes in habitat affecting species relative abundance and the invasion of new species. Invasive species could directly impact biodiversity measures through their own numbers and/or via direct competition with endemics or indirectly through habitat changes that affect both endemics and invasives. Here we measure biodiversity using several diversity indices, and examine its variation across habitat types, as well as relative to the abundance of specific invasive and/or medically important species. As an initial step to assess the impacts of biodiversity change on vector-borne diseases, we also examine the relative abundance of medically important species against habitat type and biodiversity change.

To examine patterns of mosquito diversity change we take advantage of Thailand's diversity of mosquitoes and habitat types, from highly developed to largely untouched, as well as local expertise in mosquito taxonomy and ecology. In Thailand, many mosquito borne pathogens persist despite intense eradication efforts. For example, the average number of dengue cases reported by the Department of Disease Control from 2002 to 2011 was 76,625.55 cases per year (SD = 32,983.48) (http://www.ddc.moph.go.th/), and its true disease burden is largely underestimated [18]. Chikungunya, transmitted by the same vectors as dengue, has long been ignored until recently with the advent of major outbreaks in South East Asia, including Thailand in 2008, [19], [20]. Japanese encephalitis (JE) virus, despite a vaccine program initiated in 2000, remains an important cause of encephalitis in Thailand, responsible for an estimated 15% of hospitalized encephalitis cases [21]. Malaria is also still one of the most important infectious diseases in Thailand: in spite of decades of successful control programs and dramatic reductions in the numbers of cases in most major cities, malaria remains prevalent in undeveloped rural villages and mountainous areas of Thailand [22].

Mosquito communities and the vertebrates they feed upon are an important factor in the distribution of these and other infectious diseases, yet their composition is poorly known in most areas. Studies on mosquito communities in Thailand have mainly focused on either medically important genera such as *Aedes* spp. [23], [24], [25] or specific habitats such as rice fields [26], [27], swamp forests [28], and rural villages [25]. A thorough literature search did not reveal any studies that have investigated the diversity of mosquito communities, the relative abundances of vectors, and their vertebrate communities across habitats in Thailand. In this study, we describe mosquito community diversity specifically across habitat types experiencing different levels of anthropogenic ecological pressures in central Thailand. We explore the relationship between mosquito vector relative abundance and the ecological characteristics of habitats. Ultimately, mosquito and host distribution and diversity can affect vector behaviour and vector-borne disease risk. Understanding vector community dynamics in the face of anthropogenic changes could form the basis for understanding the emergence and persistence of mosquito borne diseases.

## Methods

### Development and Characterization of a Forest-Agro-Urban Landscape Gradient

**Figure 1:**
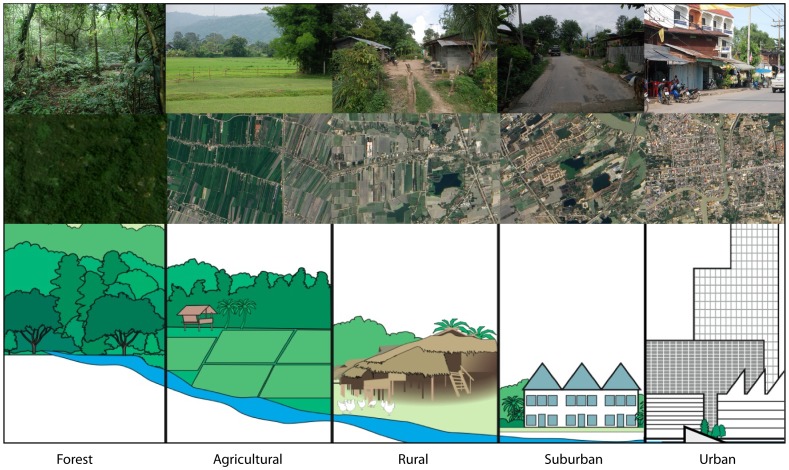
Habitat degradation gradient. Habitats found in central Thailand (top; photos by PT) represent landscape types with increasing degrees of anthropogenic modification (bottom, from left to right; drawings by Nancy Hulbirt, SOEST Illustrations) and biodiversity loss of flora and fauna, as seen by remote imaging (middle; images from NASA's Earth Observatory). Left to right: forest habitats with high biodiversity; agricultural habitats with mixed farming and forest patches to monocultures; rural habitats with some human dwellings, family farming and forest patches; suburban habitats with more human dwellings, some commercial activity, and fewer forest patches; urban habitats with dense residential and commercial activities and little to no forest patches.

**Figure 2:**
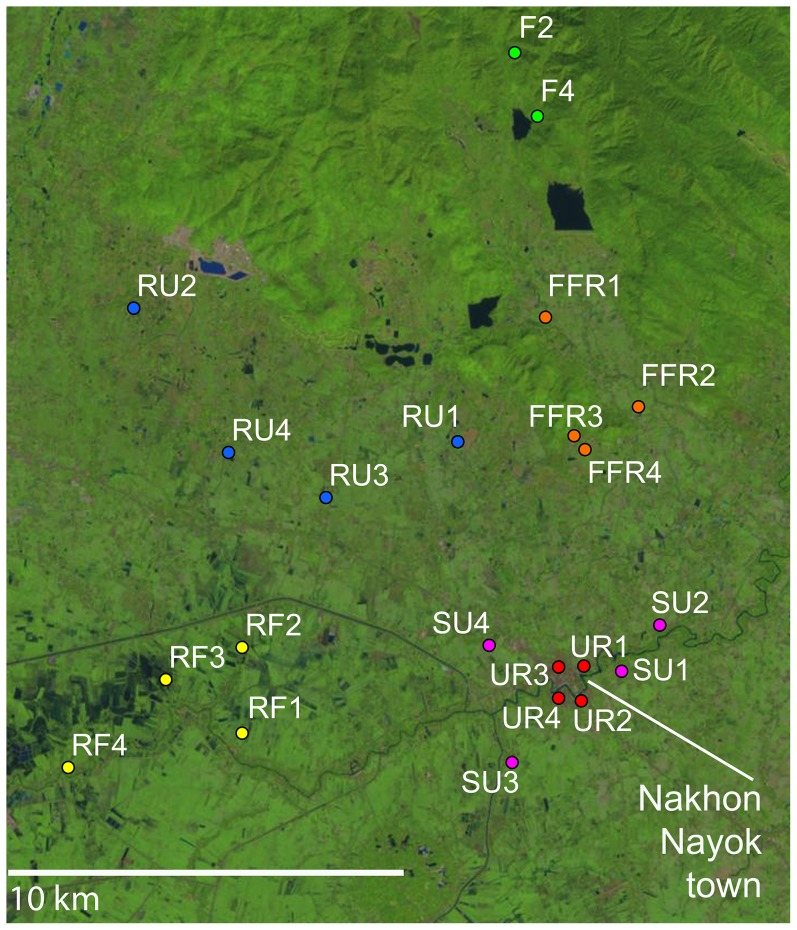
Map of study area in Nakhon Nayok Province, Thailand. Mosquitoes were collected in 24 sites representing six habitat types: Forest (F1 to F4), Fragmented Forest (FFR1 to FFR4), Rice Field (RF1 to RF4), Rural (RU1 to RU4), Suburban (SU1 to SU4), and Urban habitats (UR1 to UR2). Satellite imagery courtesy of the U.S. Geological Survey Land Remote Sensing Program (Landsat 8).

We studied six habitat types (forest, fragmented forest, rice field, rural, suburban, and urban) along a forest-agro-urban landscape gradient (Figure 1) in Nakhon Nayok province, central Thailand. Nakhon Nayok served as a suitable area for developing the gradient of habitats since the north end of the province is a part of Khao Yai National Park and the Sankambeng Range while the center of the province is a flat river plain formed by the Nakhon Nayok River and includes agricultural activities as well as more densely populated sections. The habitat types were identified along the landscape gradient first by distant imaging (Google Earth, http://www.google.com/earth/index.html) and later by direct observation. Selection criteria included the presence of human settlement, degree of human activity, degree of agricultural activity, and the amount of trash or clutter (Table 1). Within each habitat type, four sites were selected as replicates based on their similarities under these criteria (Table 1, Figure 2).

**Table 1:** Locations, trapping dates, and habitat features for 24 sites representing urban, suburban, rural, rice field, fragmented forest, and forest habitat type.

Habitat type/Site	Latitude	Longitude	Distance (km) to UR1	Trapping Dates^a^	Human Settlement^b^	Rice Field at Site	Traffic (Human/Cars)^c^	Trash/Clutter	Surrounding Area^d^
Forest - *Forest with primary growth characteristics, few signs of human disturbance*									
F1	NA	NA	NA	6/9–6/10	Absent	Absent	0/0	None	Forest
F2	N 14° 21.304	E 101° 12.127	16.58	7/2–7/3	Absent	Absent	0/0	None	Forest
F3	NA	NA	NA	6/10–6/12	Absent	Absent	0/0	None	Forest
F4	N 14° 20.150	E 101° 12.540	14.4	7/3–7/4	Absent	Absent	0/0	None	Forest
Fragmented Forest - *Secondary forest, some human dwellings with associated forest patches and/or rice field patches*									
FFR1	N 14° 17.203	E 101° 12.492	8.99	6/13–6/14	2 Houses	Present	0/0	Low	Rice field, secondary forest
FFR2	N 14° 16.067	E 101° 13.536	6.88	6/14–6/15	1 House	Absent	0/0	Low	Rice field, secondary forest
FFR3	N 14° 15.679	E 101° 12.553	6.19	7/5–7/6	1 House	Present	5/3	Low	Rice field, secondary forest, human settlements
FFR4	N 14° 15.463	E 101° 12.647	5.77	7/6–7/7	1 House	Present	NA/NA	Low	Rice field, secondary forest, human settlements
Rice field - *Continuous/multiple rice fields, other agricultural activities, trees and brush around houses*									
RF1	N 14° 11.316	E 101° 07.941	9.47	6/25–6/26	3 Houses	Present	3/19	Low	Rice field, human settlements
RF2	N 14° 12.250	E 101° 07.814	9.5	6/26–6/27	3 Houses	Present	0/14	Low/Medium	Rice field, orchard, human settlements
RF3	N 14° 12.007	E 101° 06.944	11.08	7/17–7/18	1 House	Present	14/17	Low	Rice field, orchard, human settlements
RF4	N 14° 11.073	E 101° 05.372	14.09	7/18–7/19	1 House	Present	17/32	Low	Rice field, orchard, human settlements
Rural - *Disturbed patches of vegetation, minor agriculture, trees and bushes around houses*									
RU1	N 14° 15.827	E 101° 11.157	7.28	6/19–6/20	9 Houses	Absent	1/4	Medium	Rice field, vegetation patches, human settlements
RU2	N 14° 17.799	E 101° 06.884	15.01	6/20–6/21	12 Houses	Absent	6/10	Medium	Rice field, orchard, human settlements
RU3	N 14° 15.187	E 101° 08.940	9.11	7/11–7/12	7 Houses	Absent	4/5	Low	Rice field, orchard, vegetation patch, human settlements
RU4	N 14° 15.510	E 101° 07.788	11.17	7/12–7/13	5 Houses	Present	2/1	Medium/Low	Rice field, orchard, human settlements
Suburban – *Vegetation includes mainly trees and bushes around houses*									
SU1	N 14° 12.238	E 101° 13.692	1.1	6/16–6/17	10 Houses	Absent	36/NA	High	Rice field, human settlements
SU2	N 14° 12.892	E 101° 14.244	2.29	6/17–6/18	11 Houses	Absent	24/70	Medium/High	Rice field, human settlements
SU3	N 14° 10.821	E 101° 11.922	3.54	7/7–7/9	8 Houses	Absent	7/62	Medium	Rice field, human settlements
SU4	N 14° 12.754	E 101° 12.021	2.06	7/9–7/10	18 Houses	Absent	4/15	Medium	Rice field, human settlements
Urban – *Minimal vegetation, restricted to trees and bushes around houses*									
UR1	N 14° 12.362	E 101° 13.094	0	6/22–6/23	24 Houses	Absent	48/127	High	Human settlements
UR2	N 14° 11.811	E 101° 13.037	1.02	6/23–6/24	32 Houses	Absent	89/561	High	Human settlements
UR3	N 14° 12.395	E 101° 12.785	0.56	7/14–7/15	25 Houses	Absent	51/96	High	Human settlements
UR4	N 14° 11.899	E 101° 12.749	1.06	7/15–7/16	21 Houses	Absent	22/211	Medium/High	Human settlements

a Mosquitoes were collected in the rainy season of 2008.

b Human settlement is quantified using the numbers of residential buildings in the study site (around 1,000 m×1,000 m).

c Traffic is quantified using the numbers of humans and automobiles that travel into/pass the site around noon on one of the weekdays during the 30 minutes observation periods.

d Surrounding area is assessed from the margin of the study site out to approximately 100 meters.

We trapped each site using the same combination of adult mosquito traps, designed to maximize the breadth of species encountered (see below). For each trapping session, defined as the deployment of all traps at the same site on a given date, sites were characterized for the following variables in order to quantify the level of human activity: intensity of human settlement (number of houses in the site), intensity of agricultural practice (estimated percentage of site area allotted for agricultural purposes), amount of traffic (numbers of cars and people passing by the site in 30 minutes near noon on a weekday), type of vegetation, estimated percentage of site covered by vegetation, estimated amount of trash and clutter found in the site but outside of the houses (three categories: low, medium, or high), and description of surrounding habitat (within a 100 meters radius). Other variables that may affect trap performance such as the distance of light traps from other closest artificial light sources, positioning and height of all traps, and shade cast above the traps were also collected. All environmental variables were described independently by the same two observers for all sites.

### Adult Mosquito Sampling and Identification

Mosquito collections were conducted during the rainy season of 2008 (Table 1). Four types of adult mosquito traps were used in order to maximize the variety of mosquitoes captured: the BG sentinel targets resting adults near human habitations, the Mosquito Magnet© targets host-seeking females and their attendant males, the CDC UV light trap targets nocturnally active mosquitoes of both genders, and the CDC backpack aspirator uses direct suction and was applied to potential roosts. The area trapped at each site was approximately 1 ha. The number and placement configuration of the traps, as well as the duration of sampling, were kept constant across all sites. At each site, two Mosquito Magnets© were placed in desirable locations 50 meters apart, four CDC UV light traps and four BG sentinel traps were placed 10–20 meters from each magnet. All traps were at least 10 meters away from each other. CDC UV light traps were hung outdoors from tree branches or other structures and situated 1.5 to 2 meters from the ground. If a residence was present at the site, permission was requested verbally to use the BG sentinel trap and aspirator in and around the dwelling. All residents readily gave permission and were enthusiastic to have mosquitoes removed from their vicinity. No data on humans or identifiable data to link individuals with survey results were collected, including locality data, which refers to a centralized location unlinked to a specific residence. Mosquitoes were collected for 24 hours per trapping session, with different trapping regimes for day and night. Day trapping, from 10 am to 6 pm, consisted of eight BG sentinels, two Mosquito Magnets©, and three sessions of 3–10 minute long aspirations. Night trapping, from 6 pm to 10 am, consisted of eight BG sentinels, two Mosquito Magnets©, and eight UV light traps. Thus the only difference between day and night trapping regimes was the replacement of aspiration sessions in the day with UV light traps at night. The timing of the trapping sessions at replicate sites was designed so that mosquitoes from at least two sites of the same habitat type were collected one day apart (Table 1). The trap contents were collected in the evening and in the morning and transported on ice to the field base where mosquitoes were separated on a chill table from other insects and stored at −20°C.

Mosquito samples were then transported to the laboratory at Mahidol University, where males were separated out and female mosquitoes were identified using available morphological keys [29], [30], [31], [32], [33], [34] with assistance from Thai mosquito expert Dr. Rampa Rattanarithikul. Dr. Rampa trained two graduate students (PT and AG) and one technician to assist with the identification process, which took several months at 24–32 person hours per day. Identification keys used followed the taxonomic nomenclature of Knight and Stone [35] and supplements to that publication. However, in the last 10 years there have been major revisions of tribe Aedini (Neveu-Lemaire), including the formal recognition of 80 genera within the tribe [36]. Although the identification keys reflect this reclassification, we maintained usage of the traditional taxonomic names: our diversity indices remain the same using either taxonomic scheme, and the use of traditional nomenclature should avoid confusion and communication difficulties among researchers and the general public. When species identification was not possible, specimens were grouped together in higher taxonomic categories (genus or family). Males, partial specimens or those in bad condition such that they were unidentifiable were excluded from further analysis. Three to twenty specimens were pinned and archived as vouchered reference specimens for each taxon identified. The archived specimens are housed at the Center of Excellence for Vectors and Vector-borne Diseases, Mahidol University, Thailand.

### Data Analysis

Statistical analyses were performed in R version 2.13.0 (2011, The R Foundation for Statistical Computing, http://www.R-project.org). Total abundances of male and female mosquitoes and the mean numbers of mosquitoes captured indoors and outdoors per trap were calculated across all sites and averaged for each habitat type. To test for habitat effects, the Kruskal-Wallis one-way analysis of variance by ranks was used to compare the average abundances between habitat types. The differences between the mean numbers of mosquitoes captured indoors and outdoors per trap in each habitat were compared using the Wilcoxon-Mann-Whitney rank sum test.

Mosquito diversity between habitat types was assessed by combining measures of species richness (number of species or taxa) and heterogeneity (number of species and their relative abundance). Because of the differences in numbers of mosquitoes collected at each site, species richness cannot be compared directly across habitat types. We used two strategies to correct for unequal sample size: 1) individual-based rarefaction, which allows the calculation of species richness for a given number of sampled individuals (species density or S_D_), and 2) non-parametric extrapolation-based estimation, which extrapolates species accumulation curves and estimates ‘true’ species richness based on the number of rare species in the sample. Rarefaction-based estimates and their 95% confidence intervals (CIs) for all sites were computed using the function *rarefy* in R (‘vegan’ package). Individual-based rarefaction curves for all sites were constructed from software EstimateS. Two estimators of the ‘true’ number of species in each site, Chao1 and ACE (Abundance-base Coverage Estimator), were calculated using the command estimateR in the ‘vegan’ package. Shannon and Simpson diversity indices were used as a measure of community heterogeneity.

As a first step to assessing the impacts of biodiversity change specifically on vector-borne diseases, we examine the relative abundance of specific species that act as disease vectors against habitat type and biodiversity change. Average abundance of important vector species, including invasives, was characterized for each habitat type, and statistically assessed for significance using ANOVA. Correlation analysis (Pearson's test) was used to assess the significance of the relationship between the proportion of a given species, and its abundance, log-transformed, relative to mosquito community diversity indices (Chao1 and ACE). For all statistical analysis, significance was considered if *p*<0.05.

Biodiversity of mosquito communities may change across landscapes through multiple mechanisms, including changes in habitat affecting species relative abundance and the invasion of new species. Invasive species could directly impact biodiversity measures through their own numbers and/or by interacting with endemics via direct competition or indirectly through habitat changes that disproportionately affect both endemics and invasives (e.g. the presence of insecticide to which invasive species have greater resistance). To determine to what degree biodiversity change in mosquito communities is 1) a direct result of the addition of invasives (addition of invasive species drives the value of biodiversity indices), or 2) a result of invasive/endemic species interactions or habitat change, we compare biodiversity index ACE to the relative abundance of specific invasive and/or medically important species using indices generated on all species at a given site, and indices generated without the specific invasive included. We call the latter the residual biodiversity index. If variation in residual biodiversity index ACE is correlated with a given vector's abundance, this suggests that the species is either interacting with the other species directly (e.g. competition) and/or its abundance reflects habitat changes that differentially affect all species above and beyond the impact of their numbers on the generation of biodiversity measures (e.g. mechanism 2 above).

## Results

### Forest-Agro-Urban Landscape Gradient

Mosquitoes were collected along a forest-agro-urban landscape gradient in Nakhon Nayok province, central Thailand (Figure 2). The latitude and longitude for 24 sites representing six habitat types, and their habitat characteristics, are listed in Table 1. Forest sites were characterized by primary growth and no human impacts and were situated along the border of Khao Yai National Park and at least 7 km from human settlements. No agricultural lands and domestic vertebrates were present in these sites or nearby. Fragmented forest sites were situated on the edge of a secondary forest patch fragmented from the National Park where human settlements were sparse (1–2 houses within each site) and where small scale, mixed, and non-irrigated agriculture was practiced. Most farmers in these sites either used water buffalo for pulling farming equipment or as a status symbol. The rice field sites were in the lowland closer to the Nakhon Nayok River to facilitate irrigated rice agriculture. Here, the use of water buffalo was replaced by industrialized machinery. Large continuous rice fields and small orchards could be found surrounding the farmers' houses. The rural, suburban, and urban sites were distributed based on the distance from the center of town. The urban sites were near the center of Nakhon Nayok town where agricultural settings and large natural vegetation patches were absent. In the urban sites, the numbers of houses (average 25.5±4.65 houses per site), human traffic (average 52.5±27.6 people walked into/past the site during the 30-minute observation period), car traffic (average 248.75±213.76 automobiles were driven into/past the site during the observation period), amount of trash and clutter (categorized as medium/high or high) was highest. Vegetation patches and/or landscaping was found around some houses and in empty lots. Suburban sites were 1–3 km from the town center. Houses were arranged in rows or clusters along the main paved street with an average of 11.75 houses per site (±4.35). Average number of people (17.75, ±15.02) and automobile (49, ±29.72) traffic in the site were in between the urban and rural sites. The amount of trash and clutter in the suburban sites was either medium or high. There were small patches of active rice fields and empty vegetated lots surrounded the sites. Rural sites were between 7 to 15 km from the town center. Houses were arranged in clusters with an average of 8.25 houses per site (±2.98). The house clusters were situated next to either agricultural land such as rice fields and orchards or secondary forest. Rural sites had the lowest amount of trash and clutter (categorized as low or low/medium) and lowest human and car traffic of all the human residential sites (3.25, ±2.22 and 5.00, ±3.74, respectively). All sites were situated at least 0.5 km away from each other.

### Mosquito Abundance

**Figure 3:**
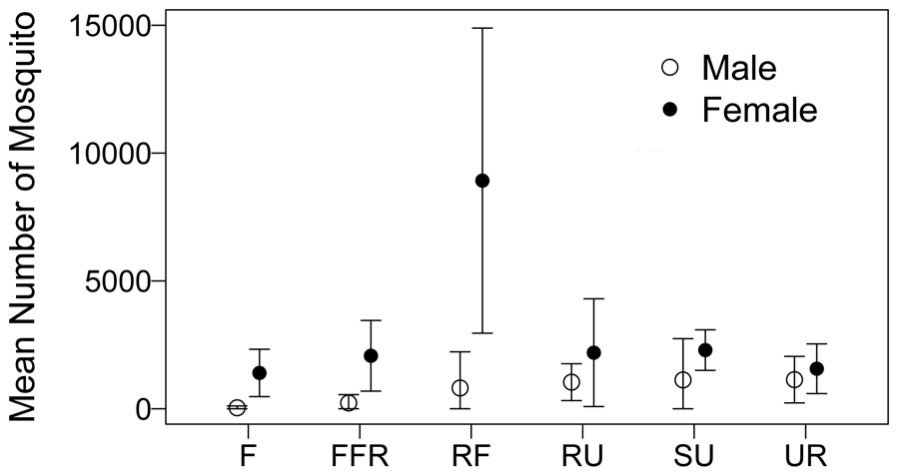
Mean abundance and 95% confidence intervals of female and male mosquitoes. Mosquitoes were caught in the forest (F), fragmented forest (FFR), rice field (RF), rural, (RU) suburban (SU), and urban (UR) habitats in Nakhon Nayok Province, Central Thailand, during the rainy season of 2008. Each habitat type is represented by four replicate sites, except for the rice field habitat where only three sites were included in the analysis.

A total of 83,325 mosquitoes was collected over the six-week period from 24 sites representing the six habitat types described. The total numbers of mosquitoes caught were significantly different among habitats (Kruskal-Wallis, chi-squared = 13.2, df = 5, P = 0.0213). The highest number of female mosquitoes was caught in the rice field habitat and the average abundance was 8,922 mosquitoes caught within 24 hours per site (sd = 2402, number of sites = 3). The lowest number of female mosquitoes was caught in the forest habitat (1,402 mosquitoes per site, sd = 582, number of sites = 4). The average abundance of male and female mosquitoes in each habitat type is shown in Figure 3. Out of all mosquitoes captured, 62,126 female mosquitoes could be morphologically identified into 109 taxa spanning 15 genera. Of these, 27,013 individuals were further classified into 68 species. The remaining 35,113 individuals were only identified to genus, subgenus, group, or subgroup either because specimens were damaged in the trap and/or they belonged to cryptic species complexes. Mosquito identifications were overseen and verified by expert SE Asian taxonomist Dr. Rampa. Taxa and their abundance by habitat over the trapping period are listed in Table S1.

The most dominant taxa overall were the *Culex (Culex)* spp. of the Vishnui subgroup (n = 28,967 or 46.63% of all identifiable female mosquitoes), *Cx. (Cux.) gelidus* (Theobald) (n = 6,246 or 10.05%), *Cx. (Oculeomyia) sinensis* (Theobald) (n = 4,261 or 6.86%), and *Cx. (Cux.) quinquefasciatus* (Say) (n = 3,535, or 5.69%). The *Culex* spp. of the Vishnui subgroup was also the most dominant taxon in the fragmented forest habitat (n = 4,238 or 53.77% from fragmented forest), rice field habitat (n = 17,853 or 65.92%), rural habitat (n = 2,659 or 34.01%), and suburban habitat (n = 3,011 or 34.77%). The most abundant taxon for the forest habitat was *Uranotaenia* spp. (n = 2,694 or 56.68%) and for the urban habitat, *Cx. quinquefasciatus* (n = 1,839 or 31.01%).

**Figure 4:**
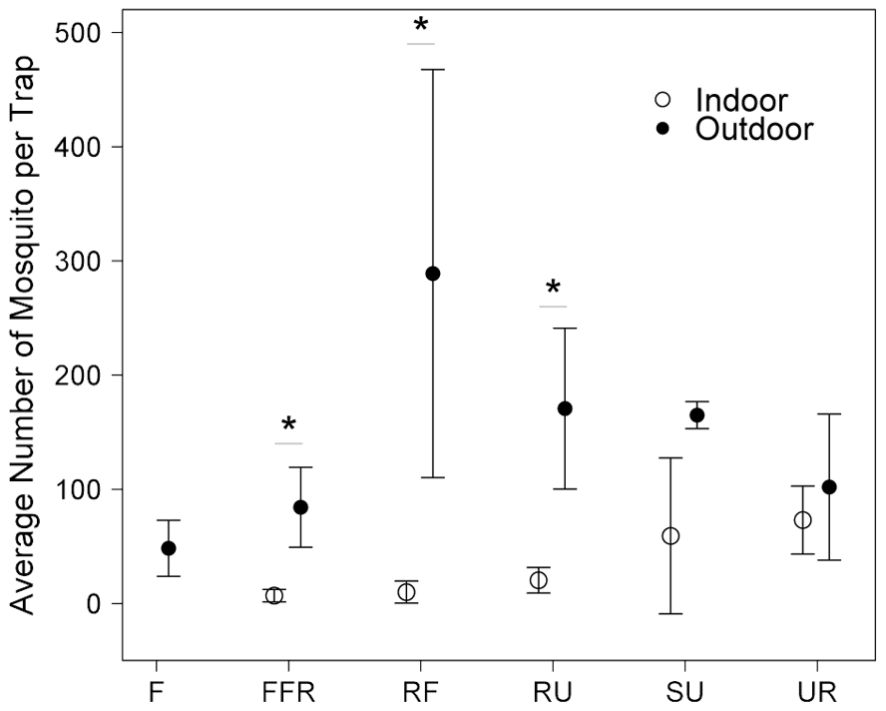
Average number of mosquitoes caught indoors and outdoors per trap and 95% confidence intervals. Mosquitoes were caught in the forest (F), fragmented forest (FFR), rice field (RF), rural (RU), suburban (SU), and urban (UR) habitats in Nakhon Nayok Province, Central Thailand, during the rainy season of 2008. Each habitat type is represented by four replicate sites, except for the rice field habitat where only three sites were included in the analysis. Wilcoxon-Mann-Whitney rank sum test was used in the analysis. Stars indicates P = 0.029. P-value for the suburban and urban habitat were 0.057 and 0.686, respectively.

Except for the forest habitat where there was no indoor trapping, the only two habitats in which the number of mosquitoes caught outdoors per trap was not significantly higher than the number of mosquitoes caught indoors per trap were the suburban and urban habitats (Wilcoxon-Mann-Whitney test; Figure 4). The average number of mosquitoes caught outdoors per trap was highest in the rice field habitat. The highest rate of indoor trapping was found in the urban habitat. The most abundant species indoors was *Aedes (Stegomyia) aegypti* (Linnaeus) in the rural (n = 210 or 39.85% of all mosquitoes collected indoors in the rural habitat), and rice field habitats (n = 31, 44.29%), *Culex* spp. of the Vishnui subgroup in the fragmented forest (n = 20, 28.17%), and *Cx. quinquefasciatus* in the suburban (n = 977, 81.48%) and urban habitats (n = 851, 67.43%).

### Diversity Indices, Richness and Rarefaction Curves

The average numbers of taxa identified (N), Shannon diversity (H), Simpson diversity (D), Chao1, and ACE indices generally varied significantly across habitat type (Table 2). The average diversity indices for the six habitat types ranged from 1.21 to 2.30 for Shannon index and from 0.51 to 0.82 for Simpson index. Both indices were highest in the rural habitat and lowest in the rice field habitat. Analysis of variance (ANOVA) tests revealed significant differences in both diversity indices among the six habitat types (F = 5.68, df = 5, P = 0.0029 for Shannon index and F = 4.50, df = 5, P = 0.0086 for Simpson index). Tukey multiple comparisons of means revealed significant differences in Shannon indices between rural-forest (P = 0.0158), rural-fragmented forest (P = 0.0452), rural-rice field (P = 0.0030), and urban-rice field (P = 0.0416) and in Simpson indices between rural-rice field (P = 0.0177).

**Table 2:** Mean species richness and diversity indices (±95% Confidence Interval) of mosquito communities found in six habitat types of Nakhon Nayok Province, Central Thailand, in 2008.

Habitat Type	N^a^	Species Density	Shannon Index	Simpson Index	Chao1	ACE
Forest	4	21.62 (5.68)	1.47 (0.54)	0.60 (0.21)	35.56 (13.81)	39.04 (13.64)
Fragmented Forest	4	26.59 (2.71)	1.59 (0.29)	0.59 (0.10)	36.73 (4.38)	38.65 (3.78)
Rice Field	3	18.20 (3.38)	1.21 (0.06)	0.51 (0.10)	34.00 (7.78)	35.03 (7.66)
Rural	4	28.37 (3.17)	2.30 (0.09)	0.82 (0.03)	35.30 (4.51)	35.97 (3.71)
Suburban	4	23.56 (2.94)	1.80 (0.13)	0.72 (0.04)	33.31 (5.01)	32.91 (4.01)
Urban	4	22.89 (5.24)	2.00 (0.36)	0.78 (0.09)	29.14 (6.70)	30.99 (6.34)

a Number of sites or replicates for each habitat type.

**Figure 5:**
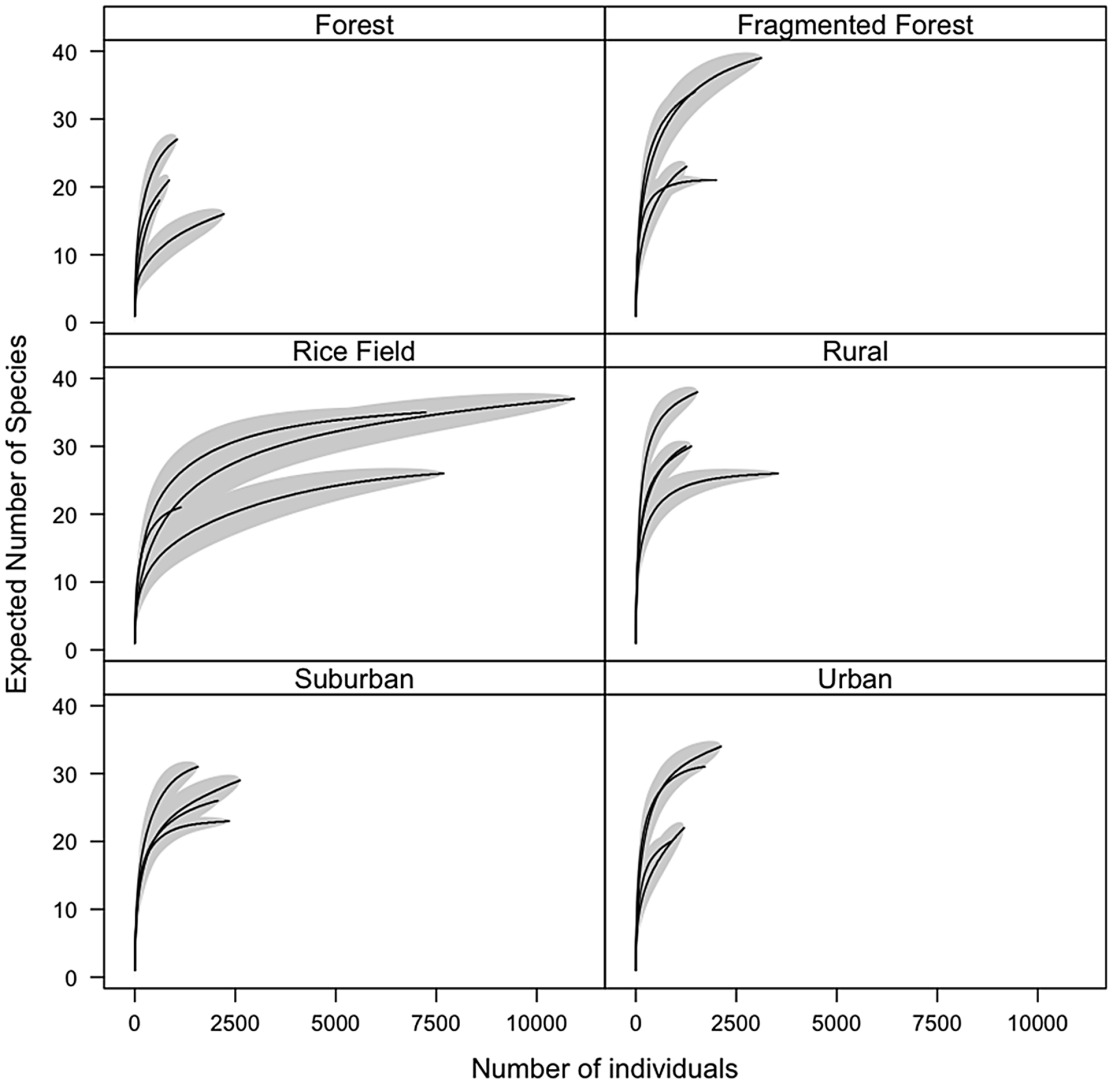
Rarefaction curves. Calculated number of mosquito taxa as a function of number of sample collected from 24 sites representing six habitat types (solid lines) and 95% confidence intervals (shaded area) were plotted. The curves are used to determine whether the number of mosquitoes collected has reached an asymptote such that 100% of possible species were sampled. The technique also allows the calculation of species richness for a rarefied number of mosquitoes (species density or S_D_).

Number of taxa identified was highest in the fragmented forest habitat (average number of taxa = 34.25), and lowest in the forest habitat (average number of taxa = 26.25). The forest site also yielded the least number of individuals, was the most difficult to sample, and this probably accounted for the lower number of species. To correct for unequal sample sizes among sites, numbers of species were rarefied to a constant number of individuals. At an equal sample size of 614, expected number of species or species density (S_D_) was highest in the rural habitat (S_D_ = 28.37, 95% CI = 25.20 to 31.53) and lowest in the rice field habitat (S_D_ = 18.20, 95% CI = 14.82 to 21.57). Individual-based rarefaction curves were constructed to determine whether the number of mosquitoes collected reached the point where species richness is saturated (Figure 5). Overall, the shape of the rarefaction curves for most sites indicated that more individuals needed to be collected for the curves to reach their plateau.

To estimate the number of rare and undetected species and add them to the observed richness, abundance-based extrapolated richness estimates such as Chao1 and ACE were calculated (Table 2). In contrast to some of the other indices, the highest richness was found in the fragmented forest habitat according to Chao1, and in the forest habitat according to ACE, followed by the rural, rice field, and suburban habitats. The lowest richness was in the urban habitat according to both estimates. However, the differences in Chao1 and ACE across sites were not significantly different based on ANOVA (F = 0.551, df = 5, P = 0.736 for Chao1 and F = 0.830, df = 5, P = 0.545).

### Vectors and Habitat Types

Because of the known presence of several vector-borne infectious diseases in the study area, the average abundance of important mosquito vectors was compared across habitats (Table 3). *Ae. aegypti* and *Ae. (Stg.) albopictus* (Skuse), vectors for dengue, chikungunya, and yellow fever virus, were most abundant in the urban and rural habitats, respectively. Malaria vectors, *Anopheles* spp., including the Barbirostris group, Hyrcanus group, Pyretophorus series, Neocellia series, and Neomyzomyia series, were most abundant in the rural habitat. *Mansonia* spp., which transmits *Brugia malayi*, an agent of Malayan filariasis, was most abundant in the rice field habitat. In Thailand, Bancroftian filariasis, filarial infection with the nematode *Wuchereria bancrofti*, is principally transmitted by *Cx. quinquefasciatus*. This species was found mainly in urban and suburban areas. Other possible vectors of Bancroftian filariasis include *Cx. (Ocu.) bitaeniorhyncus* (Giles), which was distributed mainly in the rice field habitat, and *Armigeres (Armigeres) subalbatus* (Coquillett), which was distributed relatively evenly across all habitat types. The principal vector of JE is *Culex* spp. of the Vishnui subgroup, which was abundant in the rice field habitat. The other possible vectors for JE include *Cx. (Cux.) fuscocephala* (Theobald), abundant in rural and fragmented forest areas, and *Cx. gelidus*, abundant in rice field and suburban areas. For all vectors whose abundance differed significantly across sites, their abundance was lowest in intact, forest sites (Table 3).

**Table 3:** Average abundance of vector species (±SE) found in the forest (F), fragmented forest (FFR), rice field (RF), rural (RU), suburban (SU), and urban (UR) habitat in Nakhon Nayok Province, Thailand in 2008.

Taxa	Habitat Type^a^						Vector for
	F	FFR	RF	RU	SU	UR	
*Aedes aegypti* Linnaeus, 1762*	0.00	4.00 (1.87)	10.00 (4.92)	58.00 (21.79)	37.00 (5.34)	72.25 (12.30)	DF, CHIK, YF
*Aedes albopictus* Skuse, 1894*	7.25 (1.49)	20.50 (7.58)	10.25 (0.48)	28.75 (3.68)	3.25 (0.75)	9.75 (2.78)	DF, CHIK, YF
*Culex* spp. - Vishnui subgroup*	12.00 (7.01)	1059.50 (331.88)	5831.33 (685.00)	664.75 (143.16)	752.75 (209.74)	289.50 (89.33)	JE
*Culex fuscocephala* Theobald, 1907	0.00	346.00 (213.09)	3.75 (0.25)	258.50 (65.96)	70.25 (28.55)	121.50 (34.21)	JE
*Culex gelidus* Theobald, 1901*	2.00 (1.15)	41.75 (19.18)	533.75 (184.30)	212.50 (134.99)	524.50 (122.50)	247.00 (60.25)	JE
*Culex quinquefasciatus* Say, 1823*	0.25 (0.25)	1.75 (0.25)	2.00 (0.82)	48.00 (23.41)	372.00 (258.63)	459.75 (74.56)	JE, Filariasis^b^
*Culex bitaeniorhynchus* Giles, 1901*	5.25 (3.32)	12.00 (5.64)	281.33 (122.64)	16.25 (3.12)	32.50 (14.51)	2.50 (1.04)	JE, Filariasis^b^
*Armigeres subalbatus* Coquillett, 1898	29.00 (15.29)	82.75 (29.54)	38.25 (20.98)	97.75 (36.52)	40.25 (7.74)	54.75 (25.62)	Filariasis^b^
*Mansonia* spp.*	1.25 (1.25)	26.50 (7.58)	312.00 (8.66)	86.50 (28.10)	139.00 (37.95)	36.75 (14.11)	Filariasis^c^
*Anopheles* spp.*	2.25 (0.85)	100.75 (44.67)	87.75 (36.66)	199.75 (80.61)	34.50 (5.55)	56.75 (24.97)	Malaria

JE: Japanese Encephalitis, DF: Dengue Fever, CHIK: Chikunkunya, YF: Yellow Fever.

a Number of site for each habitat type is 3 except for the rice field habitat which only three sites were used in the analysis.

b nematode *Wuchereria bancrofti*.

c nematode *Brugia malayi*.

* significant variation across sites according to ANOVA, p<0.05 (for Anopheles spp., p<0.057).

### Vector Abundance and Mosquito Community Diversity

Correlation analysis revealed a significant negative correlation between ACE index and the fraction of *Aedes aegypti* in the total number of mosquitoes (r = −0.46, t = −2.35, df = 21, P = 0.0287) and of *Culex quinquefasciatus* (r = −0.49, t = −2.55, df = 21, P = 0.0185). This relationship was not observed with the other medically important species. To determine to what extent these relationships reflect the numerical influence of *Ae. aegypti* or *Cx. quinquefasciatus* on the statistical value of the ACE index versus inherent properties of species or habitat interactions, we examined the correlation between vector relative abundance at sites where present and the ‘residual’ diversity index calculated without that species included and found a significant negative correlation between the ‘residual’ ACE index and *Aedes aegypti* relative abundance (r = −0.52, t = −2.77, df = 21, p-value = 0.0115) and *Culex quinquefasciatus* relative abundance (r = −0.47, t = −2.44, df = 21, p-value = 0.0238). These results suggest that these vector species are found in truly reduced diversity environments, whose index is not simply a statistical consequence of the given invasive vector's abundance.

## Discussion

With recent global expansions of humans and vectors, new and recurring infectious diseases have emerged, often in epidemic proportions, and in some cases have been correlated with changes in the biodiversity of affected communities [2]. Biodiversity can increase the resilience of communities to change [37]. A hallmark of disturbed ecosystems includes the emergence of infectious diseases, which has also been correlated with biodiversity loss [2], [38]. Here we examined the relationship between mosquito diversity and habitat modification by humans across a range of sites, from primary forest to urban centers, in Central Thailand. The mosquito communities sampled included several important vectors of infectious diseases such as dengue, chikungunya, yellow fever, filariasis, and malaria. We showed that both the diversity of mosquito communities and the relative abundance of disease vectors varied by habitat, with the lowest diversity and highest abundance of certain vectors occurring in urban environments, whereas other vectors were most abundant in different habitats depending on their biology. In all cases, vectors of disease were lowest in intact forest habitats.

We combined both a unique field design and analytical approach to explore the relationship between habitat degradation and mosquito biodiversity. We fully characterized 24 sites to represent six habitat types that varied by degree of human impact, from least to most: forest, forest fragment, agricultural, rural, suburban, and urban. Our analytical approach applied a combination of standard diversity indices as well as estimates of true richness that take into account sample size variation. To date, most studies on mosquito communities have compared the numbers of species found in communities without considering 1) the differences in the numbers of samples collected, and 2) whether sampling was sufficient to capture most species, such that species accumulation curves reached their asymptotes [39], [40], [41], [42], [43]. The number of individuals that must be sampled to reach this asymptote can be prohibitively large especially in the tropics, where species diversity is high and most species are rare [44]. Consequently, collecting enough samples is often difficult or technically impossible, and using true richness estimators is preferred. Our results indicated that estimates of true richness (Chao1 and ACE) can differ greatly from standard diversity indices (species abundance, Shannon and Simpson).

Over the six habitat types surveyed in Central Thailand, we collected 83,325 mosquitoes, of which 62,126 were females and identifiable into 109 taxa including 15 genera and 68 species. Mosquito diversity varied greatly by habitat. According to the true richness estimators, the least diverse habitats were the urban, followed by suburban, rice field, and rural. The most diverse habitats were the forest and the fragmented forest. Forest estimates of diversity are conservative and probably underestimate the diversity more than other sites. Common forest species, such as the genus *Uranotaenia*, are particularly small and difficult to identify. In addition, compared to other habitat types, the adverse conditions of the forest habitat such as more rain and humidity differentially degrades mosquito condition, possibly affecting mosquito estimates of richness compared with other sites. Fewer individuals were also collected at forest sites, suggesting a combination of lower abundance and that the habitat may be harder to survey with our trap sets, possibly due to the abundance of alternative microniches. Even under these less than ideal conditions, richness estimators still indicated that urban/suburban habitats are less diverse in terms of mosquitoes than forest/fragmented forest habitats.

Other studies that have compared mosquito communities across human-modified landscapes focused on urban, semi-urban, and rural environments also showed that urban environments are the least diverse [39], [45], [46], [47]. The mechanisms underlying this pattern are not well understood, but some have suggested the positive effect of habitat diversity on mosquito species diversity [48], [49], or that increased stress and pollution in urban habitats favor certain invasive genera such as *Culex*, which is more adaptable to a variety of habitats and may competitively exclude other species [50], [51], [52]. We suspect that the urban environments in our study may have had fewer kinds of aquatic habitats that different female mosquitoes could exploit, thus favoring human-adapted mosquitoes such as *Aedes aegypti* and *Culex quinquefasciatus*. In addition, the contamination of pesticide in households and agricultural land may alter natural aquatic community composition, influence larval mosquito abundance, and favor species that are more resistant to chemicals [53], [54].

Agricultural environments characterized by monoculture are similarly niche-poor and liable to suffer biodiversity loss. We observed that the rice field habitat, where irrigated and intensive rice cultivation is practiced, was less diverse than the rural and fragmented forest habitats, where small, non-irrigated, and mixed agriculture is practiced. A study in Kenya [40] comparing mosquito communities between planned, unplanned, and non-irrigated riceland agroecosystems found the highest diversity in non-irrigated agroecosystems and this was linked to higher habitat diversity in this environment. In our study, we observed that there were more types of aquatic habitat in rural and fragmented forest environments than in rice fields. In fact, the rural and fragmented forest habitats are ecotones, transition zones between two or more adjacent ecological systems [55], and as such should include an elevated number of species, the combined set of species from different adjacent and partially overlapping habitats. Ecotones have been shown to play a role in a number of important emerging infectious diseases [56].

The distribution of medically important mosquito species differed across habitats, correlated with biodiversity changes, and may have important implications for disease transmission in Thailand. Our results show that *Culex quinquefasciatus* was most abundant in the urban habitat both indoors and outdoors. *Cx. quinquefasciatus* uses dirty and polluted urban aquatic sources as larval habitat [50], [51], which are particularly associated with human habitation [57]. *Culex* spp. of the Vishnui subgroup, which includes the morphologically cryptic *Cx. (Cux.) vishnui* (Theobald), *Cx. (Cux.) tritaeniorhynchus* (Giles), and *Cx. (Cux.) pseudovishnui* (Colless), were found in all habitats but were most abundant in rice fields. In Thailand, *Cx. quinquefasciatus* and the Vishnui subgroup are the main vectors of filariasis and Japanese encephalitis, respectively [58], [59], [60]. *Cx. gelidus*, *Cx. bitaeniorhynchus*, and *Mansonia* spp., also vectors of filariasis and/or JE, were also most abundant in rice fields. *Anopheles* spp., vectors of malaria-causing *Plasmodium* spp., were most abundant in rural sites. *Ae. aegypti* is highly anthropophilic and prefers to feed on human blood [61] and consequently was primarily collected in urban sites, occasionally in suburban and rural sites, and rarely or never in the other habitats. *Ae. albopictus*, on the other hand, was collected primarily from rural and fragmented forest habitats, and occasionally in other habitats. These findings concur with others showing that the average number of *Ae. aegypti* was higher in urban than rural areas, whereas the opposite was found for *Ae. albopictus* [62], [63], [64]. *Ae. aegypti* and *Ae. albopictus* are important disease vectors of dengue virus, in which the former has mostly been associated with epidemic transmission [65]. All vectors were least abundant in the forest sites. Furthermore, *Ae. aegypti* and *Cx. quinquefasciatus* relative abundances were both negatively correlated with biodiversity measures across sites.

The negative relationship between mosquito abundance and site diversity for *Ae. aegypti* and *Cx. quinquefasciatus* was observed relative to both raw ACE diversity indices and the ‘residual’ indices, those derived with the direct numerical influence of each vector species removed. The relationship between vector abundance and residual diversity is a novel presentation of the data and suggests a negative interaction between these vectors and other species. Such interactions could include competition, as documented between *Ae. aegypti* and *Ae. albopictus* [66], [67], [68], [69], and *Cx. quinquefasciatus* and other *Culex* spp. [70], or that the community may be responding to a third variable affecting abundance and/or biodiversity, for example, differential habitat suitability or insecticide resistance, that disproportionally favors invasive species over native species. Although this pattern is suggestive of a potential ameliorating effect of biodiversity on human health, further studies are necessary to distinguish the causal links underlying this pattern of biodiversity change.

Evidence for the importance of biodiversity on infectious diseases in human populations is growing, yet mechanisms such as the ecological role of vectors and host communities are still controversial [3], [71]. In this study we examined patterns of mosquito community change across a range of anthropogenically-modified habitats as a first step towards identifying potential mechanisms by which vector-borne disease distribution might be affected. The result is a documentation of biodiversity change in a group seldom considered for the full breadth of its diversity. Previous mosquito studies in Thailand have been restricted to only a few habitats or important vector species, thus current knowledge of mosquito community diversity and the relative abundance of disease vectors across habitats is limited. Our patterns suggest multiple mechanisms might link biodiversity loss with human health risk across Central Thailand, including the direct invasion of specific disease-bearing vectors and their interactions with other mosquito species. Competitive interactions between key invasive vectors and other mosquitoes, such as between *Ae. aegypti* and *Ae. albopictus*, and *Cx. quinquefasciatus* and other *Culex* spp., may provide an opportunity to control the impact of anthropogenic change on invasive vector abundance.

## Supporting Information

Table S1 **Taxa and their abundance by habitat over the trapping period.**
(XLSX)
